# Metabolic syndrome, not menopause, is a risk factor for hypertension in peri-menopausal women

**DOI:** 10.1186/s40885-018-0099-z

**Published:** 2018-10-15

**Authors:** Gyu Chul Oh, Kee Soo Kang, Chan Soon Park, Ho Kyung Sung, Kyoung Hwa Ha, Hyeon Chang Kim, Sungha Park, Sang Hyun Ihm, Hae-Young Lee

**Affiliations:** 1 Department of Internal Medicine, Seoul National University College of Medicine, Seoul, Korea; 2 Department of Internal Medicine, Seoul National University Hospital, 101 Daehak-ro, Jongno-gu, Seoul, Korea; 3 Seoul National University College of Medicine, Seoul, Korea; 4 Department of Preventive Medicine, Seoul National University College of Medicine, Seoul, Korea; 5 Department of Endocrinology and Metabolism, Ajou University School of Medicine, Suwon, Korea; 6 Department of Preventive Medicine, Yonsei University College of Medicine, Seoul, Korea; 7 Department of Internal Medicine, Yonsei University College of Medicine, Seoul, Korea; 8 Department of Internal Medicine, Bucheon St. Mary’s Hospital, The Catholic University of Korea, Bucheon, Korea

**Keywords:** Hypertension, Metabolic syndrome, Menopause, Obesity

## Abstract

**Background:**

It has been long debated whether menopause itself is a risk factor for hypertension in peri-menopausal women. We aimed to assess the association between menopause and hypertension, and whether metabolic syndrome (MetS) has an influence on its effect.

**Methods:**

Data for 1502 women aged 42 to 53 from the Korean Genome and Epidemiology Study (KoGES) database were retrospectively analyzed. The KoGES database consists of 10,038 participants, of which 52.6% (5275) were female. Subjects were followed up for 4 years, and compared according to menopausal status. Additionally, 1216 non-hypertensive subjects were separately analyzed to assess whether a change in menopausal status was associated with development of hypertension.

**Results:**

The prevalence of hypertension, diabetes, and MetS for menopausal and non-menopausal subjects at baseline was 24.4% vs. 16.7%, 5.8% vs. 2.9%, and 25.4% vs. 16.6%, respectively (*p* < 0.01 for all comparisons). Among non-hypertensive subjects at baseline, prevalence of hypertension at 4-year follow-up was 9.4%, 19.7%, and 13.1% for non-menopausal, those who became menopause during follow-up, and those who were menopause at baseline, respectively. Development of hypertension was positively correlated with MetS (HR 3.90, 95% CI 2.51–6.07) and increased BMI (HR 1.09, 95% CI 1.03–1.16), while association with menopause was not significant.

**Conclusions:**

Menopause is closely associated with increased incidence of hypertension, but the increase may not be attributable to menopause itself but to increased prevalence of MetS.

**Electronic supplementary material:**

The online version of this article (10.1186/s40885-018-0099-z) contains supplementary material, which is available to authorized users.

## Background

Hypertension is a well-known risk factor of cardiovascular disease [1], and about a quarter of the global population over 20 years of age have been diagnosed [2]. The prevalence of hypertension increases with age, and women have a lower prevalence of hypertension up to the sixth decade. However, the prevalence increases rapidly as women enter the fourth and fifth decades, surpassing that of men in the sixth and seventh decades [3]. This demographic trend is observed in both Western and Asian countries [4].

Researchers have conducted studies in order to analyze the factors contributing to increased prevalence of hypertension in middle-aged women, and some have suggested menopause as a potential risk factor [5–7]. However, other studies suggest differently, that there exists no correlation between menopause and hypertension [8, 9].

Women undergo several metabolic changes near menopausal age. Hormonal changes causes the body to accumulate abdominal fat, leading to central obesity, which is a key factor of metabolic syndrome (MetS) [10, 11]. In a study using data from United States National Health and Nutrition Examination Survey (NHANES), postmenopausal status increased the risk of MetS by 60% after adjustment for age, body mass index (BMI), and other confounding variables [12]. As menopause and MetS are closely related, it is possible that the increase in prevalence of hypertension is attributable to metabolic changes related to MetS, which is prevalent in 30 to 40% of women after menopause [13, 14].

In this retrospective analysis, we sought to investigate the association between menopause and hypertension by analyzing data from the Korean Genome and Epidemiology Study (KoGES).

## Methods

### KoGES database

The KoGES cohort provides valuable evidence for prevention of major chronic diseases such as hypertension, obesity, diabetes, etc. It collects epidemiological, biological, and clinical data through questionnaires from participants aged 40 to 69, and also includes data on lifestyle, family history, and nutritional status. Clinical examinations are performed on a regular basis, and biologic specimens such as blood, urine, and DNA are collected [15]. The details of the cohort have been previously published in a separate article [15, 16].

### Study design

**Fig. 1:**
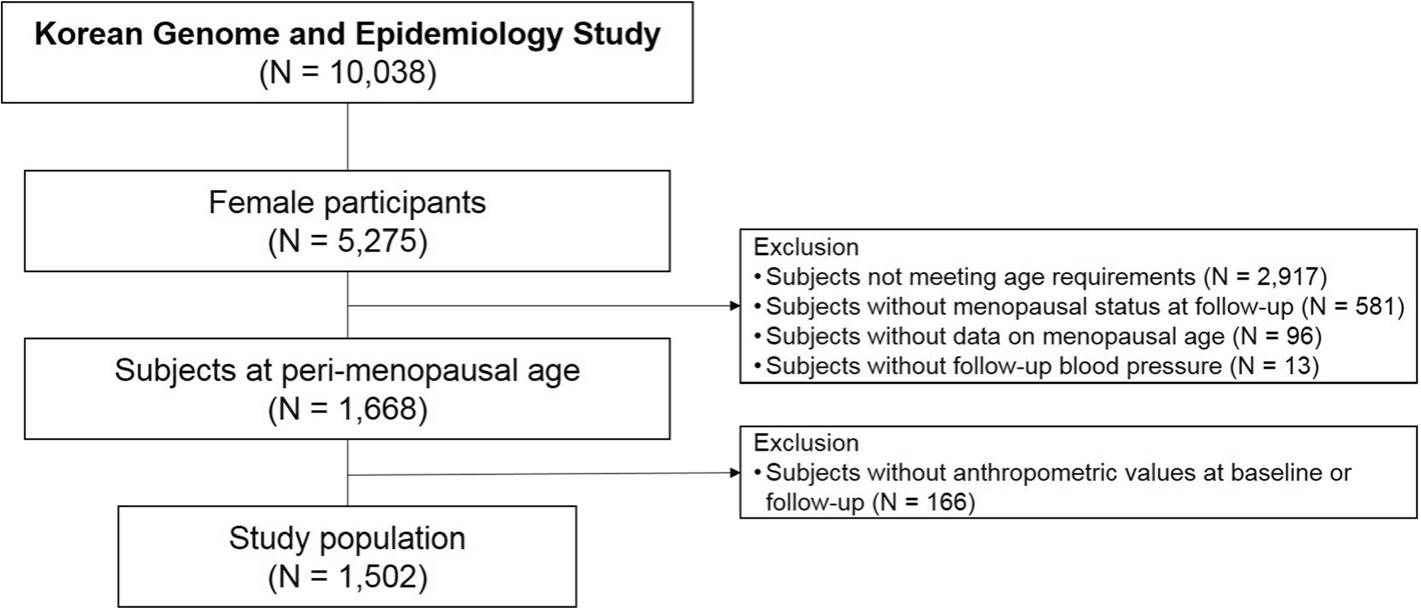
Study Design

The KoGES database included 5275 female (52.6% of 10,038) subjects at baseline. The mean menopausal age was 47.8 ± 5.2, and subjects with ages in the range of 1 standard deviation from mean (ages 42 to 53) were selected (*n* = 2345). Subjects who had not completed the 4-year follow-up were excluded, and 1502 subjects were finally analyzed for outcome. Details of the study design are provided in Fig. 1.

### Outcome analysis

The association between baseline menopausal status and development of hypertension during the study period was assessed. Risk factors for development of hypertension were analyzed and compared according to menopausal status. A subgroup of patients who were not hypertensive at baseline separately analyzed.

### Variable definitions

Blood pressure was measured in both arms using a standard mercury sphygmomanometer after resting for at least 10 min. The average of two measurements, acquired with a 5-min interval was used for analysis. Hypertension was defined as systolic blood pressure (SBP) ≥140 mmHg or diastolic blood pressure (DBP) ≥90 mmHg and prehypertension as SBP ≥120 mmHg or DBP ≥80 mmHg [17, 18]. Blood pressure was measured in the sitting position, and the average value was used. Subjects with previous history of hypertension, or those on anti-hypertensive medications were defined as hypertensive.

Diabetes mellitus was defined if the subject had at least one of the following: (i) fasting plasma glucose ≥126 mg/dL, (ii) two-hour plasma glucose ≥200 mg/dL during a 75 g oral glucose tolerance test [19], and (iii) HbA1c ≥6.5%. Subjects on insulin or oral hypoglycemic agents were also categorized as having diabetes.

MetS was defined using the modified National Cholesterol Education Program Adult Treatment Panel III (NCEP-ATP III) guideline [20, 21], with values for waist circumference modified to the Korean standard reported by the Korean Society for the Study of Obesity [22]: (i) central obesity (waist circumference > 85 cm in women), (ii) hypertriglyceridemia (fasting triglyceride ≥150 mg/dL), (iii) low high density lipoprotein-cholesterol (HDL-C) (HDL-C < 50 mg/dL for women), (iv) raised blood pressure (SBP ≥130 mmHg, DBP ≥85 mmHg, or current use of antihypertensive medications), and (v) fasting plasma glucose concentration ≥ 100 mg/dL or current use of diabetes medications.

Menopause was defined as having periods of 3 months or more without menstruation, and age at menopause was also recorded.

### Statistical analysis

Continuous variables are reported as means ± standard deviation, and categorical variables are reported as percentages of the total population. Student’s *t*-test was used for comparison of continuous variables, and one-way ANOVA or chi-square test were used for categorical variables. Univariate regression analysis was performed for non-hypertensive patients at baseline to assess risk factors associated with development of hypertension. Multivariate regression analysis using variables such as age, menopausal status, BMI, and MetS was performed. For multivariate analysis, stepwise backward elimination by likelihood ratio was conducted (probabilities of stepwise entry and removal were both 0.05). For every statistical analysis, two-sided *p* value lower than 0.05 was defined as statistically significant. All analyses were performed using IBM SPSS Statistics version 22 (SPSS Inc., Chicago, IL, USA).

### Ethics

KoGES was conducted in conformity with the Declaration of Helsinki. The institutional review boards of the Korea Centers for Disease Control and Prevention, Ajou University Hospital (IRB No. AJIRB-CRO-06-039), and Korea University Ansan Hospital (IRB No. ED0624) approved of the study. Informed consent was obtained from all subjects. This retrospective analysis using the KoGES cohort was separately approved by Seoul National University Hospital (IRB No. 1607–102-776).

## Results

### Baseline and 4-year follow-up characteristics

Among 1502 subjects, 464 were menopausal at baseline while 1038 were pre-menopause. Menopausal subjects were older (49.2 ± 3.1 vs. 45.4 ± 2.7 years, *p* < 0.001), more obese [BMI (25.0 ± 3.2 kg/m^2^ vs. 24.6 ± 3.2 kg/m^2^, *p* = 0.04), had higher values for waist circumference (81.1 ± 9.2 cm vs. 78.6 ± 8.7 cm, *P* < 0.001)], and had higher lipid profiles [TG (148.4 ± 94.2 mg/dL vs. 129.1 ± 71.4 mg/dL, *P* < 0.001), total cholesterol (195.9 ± 36.0 mg/dL vs. 182.1 ± 31.0 mg/dL, *P* < 0.001)] than non-menopausal subjects. Baseline demographic details are summarized in Table 1. At the 4-year follow-up period, 830 subjects were menopausal while 672 were not. The demographics of menopausal and non-menopausal subjects were similar to that of the baseline survey (Additional file 1: Table S1).

**Table 1:** Baseline characteristics according to menopausal status

	Non-menopause (*N* = 1038)	Menopause (*N* = 464)	*p*-value
Age (years)	45.4 ± 2.7	49.2 ± 3.1	< 0.001
Weight (kg)	59.5 ± 8.1	60.0 ± 8.3	0.262
Height (cm)	155.4 ± 5.2	154.9 ± 5.0	0.063
BMI (kg/m^2^)	24.6 ± 3.2	25.0 ± 3.2	0.040
SBP (mmHg)	114.3 ± 16.0	118.2 ± 18.0	< 0.001
DBP (mmHg)	75.9 ± 10.7	78.9 ± 11.5	< 0.001
Waist circumference (cm)	78.6 ± 8.7	81.1 ± 9.2	< 0.001
TG (mg/dL)	129.1 ± 71.4	148.4 ± 94.2	< 0.001
Total cholesterol (mg/dL)	182.1 ± 31.0	195.9 ± 36.0	< 0.001
HDL-cholesterol (mg/dL)	46.8 ± 9.8	46.2 ± 9.9	0.242
Fasting glucose (mg/dL)	81.7 ± 9.0	82.1 ± 9.1	0.499
HbA1c (%)	5.5 ± 0.4	5.6 ± 0.4	< 0.001
Alcohol consumption (%)	31.5	28.9	0.308
Hypertension prevalence (%)	16.7	24.4	< 0.001
Diabetes prevalence (%)	2.9	5.8	0.006
MetS prevalence (%)	16.6	25.4	< 0.001

*BMI* body mass index, *SBP* systolic blood pressure, *DBP* diastolic blood pressure, *TG* triglyceride, *MetS* metabolic syndrome. Data are presented as mean ± SD for continuous variables and percentages for categorical variables

### Prevalence of hypertension, diabetes mellitus, and MetS

**Fig. 2:**
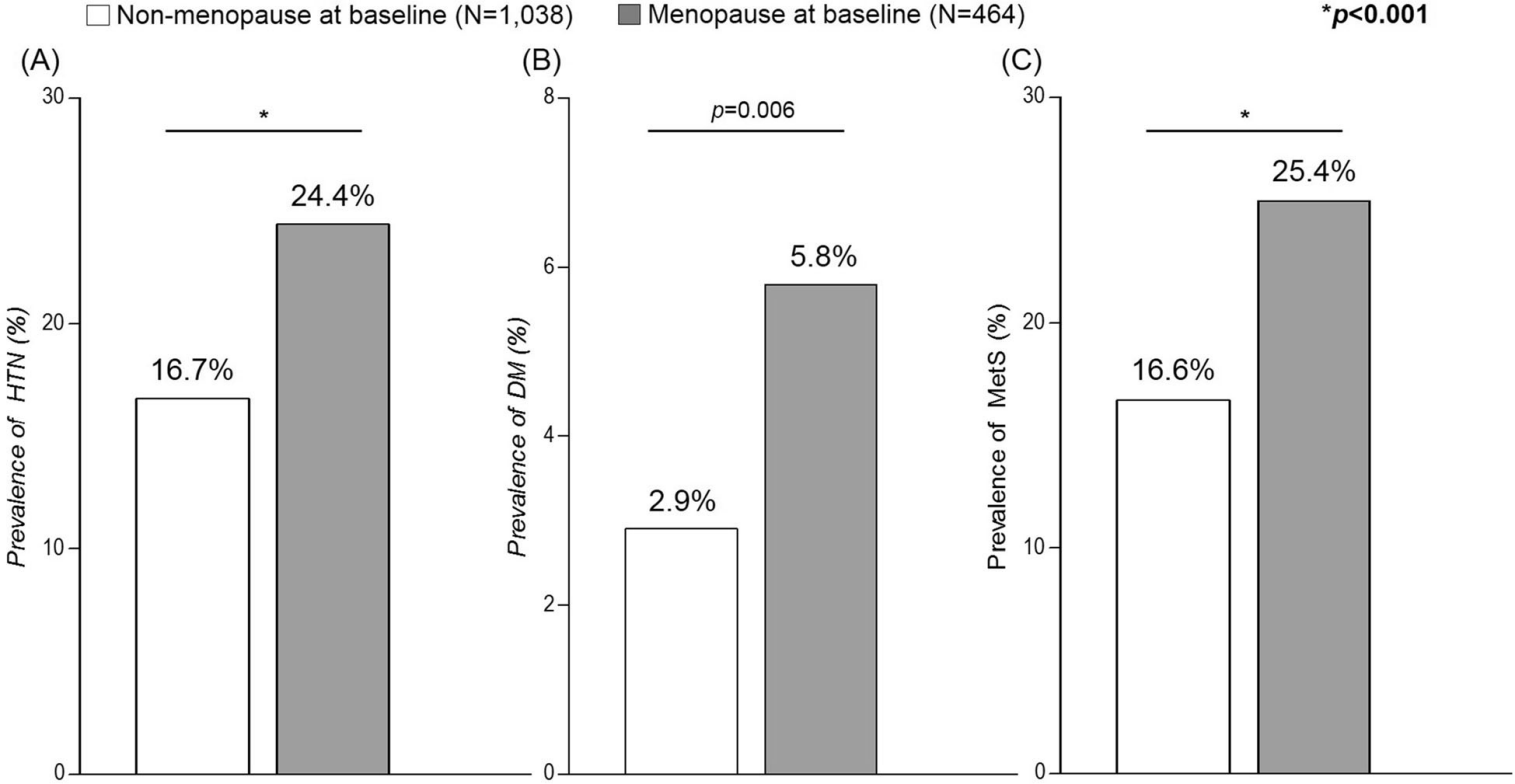
Prevalence of (**a**) hypertension, (**b**) diabetes mellitus, and (**c**) metabolic syndrome at baseline. HTN, hypertension; DM, diabetes mellitus; MetS, metabolic syndrome

**Fig. 3:**
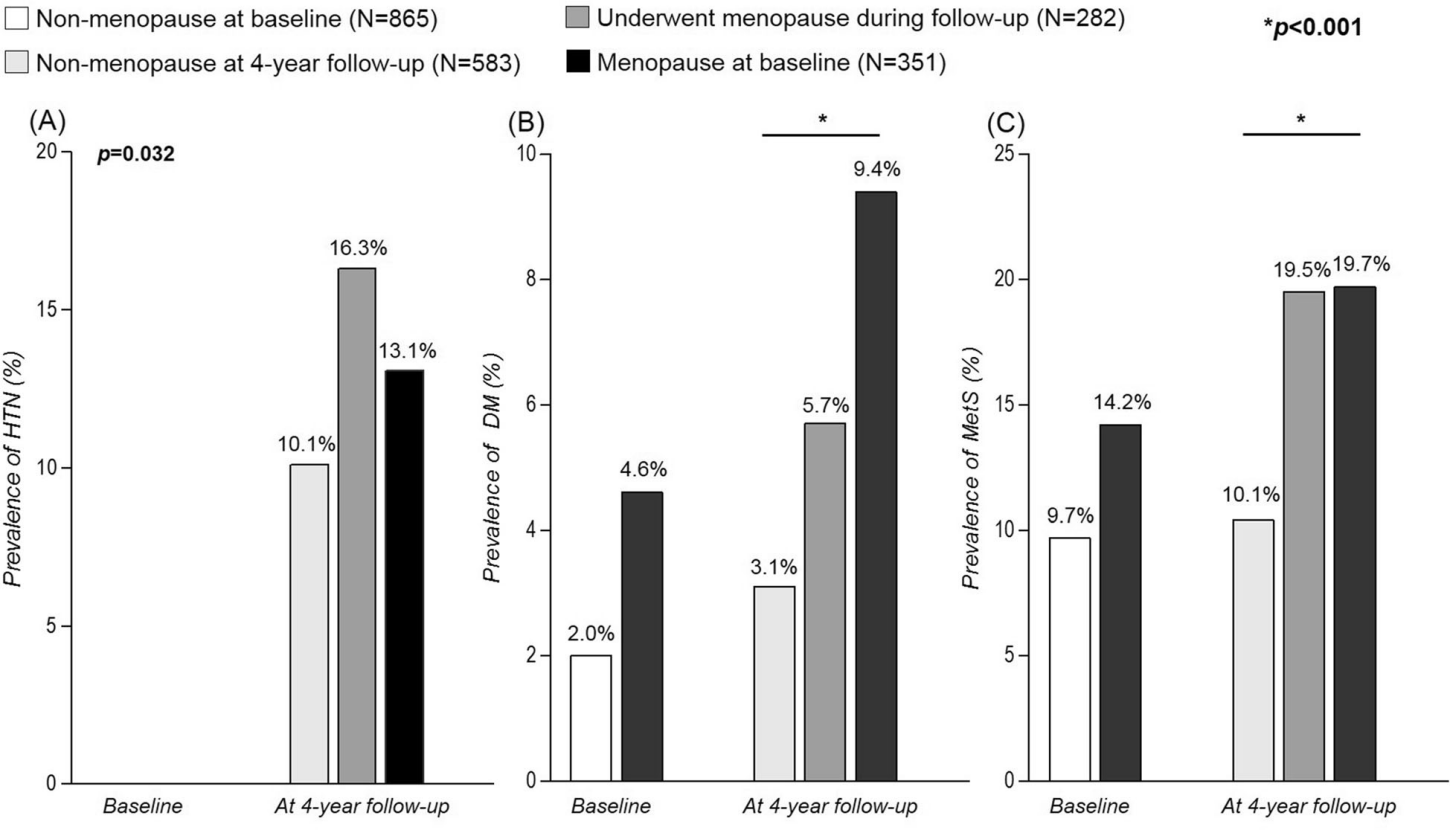
Prevalence of (**a**) hypertension, (**b**) diabetes mellitus, and (**c**) metabolic syndrome at 4-year follow-up. HTN, hypertension; DM, diabetes mellitus; MetS, metabolic syndrome

The prevalence of hypertension in menopausal and non-menopausal subjects at baseline was 24.4% and 16.7%, respectively (*P* < 0.001, Fig. 2a). Diabetes mellitus (5.8% vs. 2.9%, *P* = 0.006, Fig. 2b) and MetS (25.4% vs. 16.6%, *P* < 0.001, Fig. 2c) were also more frequent in menopausal than non-menopausal subjects at baseline. The trend of higher prevalence of hypertension, diabetes, and MetS in menopausal subjects were also consistent at the 4-year follow-up period (34.7% vs. 21.7% for hypertension, 8.7% vs. 4.0% for diabetes, and 27.6% vs. 15.0% for Mets, all *P* < 0.001) (Fig. 3).

### Subgroup analysis of non-hypertensive subjects

Subjects who were not hypertensive at baseline were separately analyzed and compared according to change in menopausal status. Among 1216 non-hypertensive subjects, 351 were menopause at baseline, and 282 subjects went through menopause during the 4-year follow up period.

Subjects who were menopause at baseline, and those who underwent menopause during follow-up had increased values for waist circumference (81.0 ± 8.2 cm, 81.5 ± 8.8 cm vs. 78.9 ± 7.9, *p* < 0.001), total cholesterol (202.7 ± 31.4 mg/dL, 201.8 ± 36.0 mg/dL vs. 189.0 ± 31.7 mg/dL, *p* < 0.001), and TG (125.9 ± 75.9 mg/dL, 127.2 ± 73.7 mg/dL vs. 103.5 ± 71.6 mg/dL, *p* < 0.001), compared to those who were non-menopausal during the study period (Table 2).

**Table 2:** Four-year follow-up characteristics of non-hypertensive subjects at baseline

	Total	Change in menopausal status			
		Non-menopause after 4 years (*N* = 583)	Menopause after 4 years (*N* = 282)	Menopause at baseline (*N* = 351)	*P*-value
Age (years)	50.1 ± 3.2	48.2 ± 1.9	51.0 ± 2.8	52.7 ± 3.2	< 0.001
Weight (kg)	58.7 ± 7.5	58.5 ± 7.4	58.7 ± 7.1	59.0 ± 8.1	0.677
Height (cm)	155.5 ± 5.2	155.7 ± 5.2	155.5 ± 5.6	155.1 ± 5.2	0.206
BMI (kg/m^2^)	24.3 ± 3.0	24.1 ± 2.9	24.3 ± 2.7	24.5 ± 3.1	0.184
Waist circumference (cm)	80.2 ± 8.3	78.9 ± 7.9	81.0 ± 8.2	81.5 ± 8.8	< 0.001
HbA1c (%)	5.4 ± 0.5	5.3 ± 0.4	5.4 ± 0.5	5.5 ± 0.6	< 0.001
Fasting glucose (mg/dL)	88.4 ± 12.3	87.2 ± 9.0	89.1 ± 13.5	89.8 ± 15.6	0.005
Total cholesterol (mg/dL)	195.8 ± 33.6	189.0 ± 31.7	202.7 ± 31.4	201.8 ± 36.0	< 0.001
HDL-cholesterol (mg/dL)	47.1 ± 10.3	47.6 ± 10.4	46.5 ± 9.5	46.8 ± 10.8	0.334
TG (mg/dL)	115.6 ± 74.1	103.5 ± 71.6	125.9 ± 75.9	127.2 ± 73.7	< 0.001
Alcohol consumption (%)	30.8	34.8	25.2	28.5	0.009
Diabetes (%)	5.5	3.1	5.7	9.4	< 0.001
MetS (%)	15.1	10.3	19.5	19.7	< 0.001
Hypertension (%)	12.9	10.1	16.3	13.1	0.032

*BMI* body mass index, *SBP* systolic blood pressure, *DBP* diastolic blood pressure, *TG* triglyceride, *MetS* metabolic syndrome. Data are presented as mean ± standard variables for continuous variables and percentages for categorical variables

**Fig. 4:**
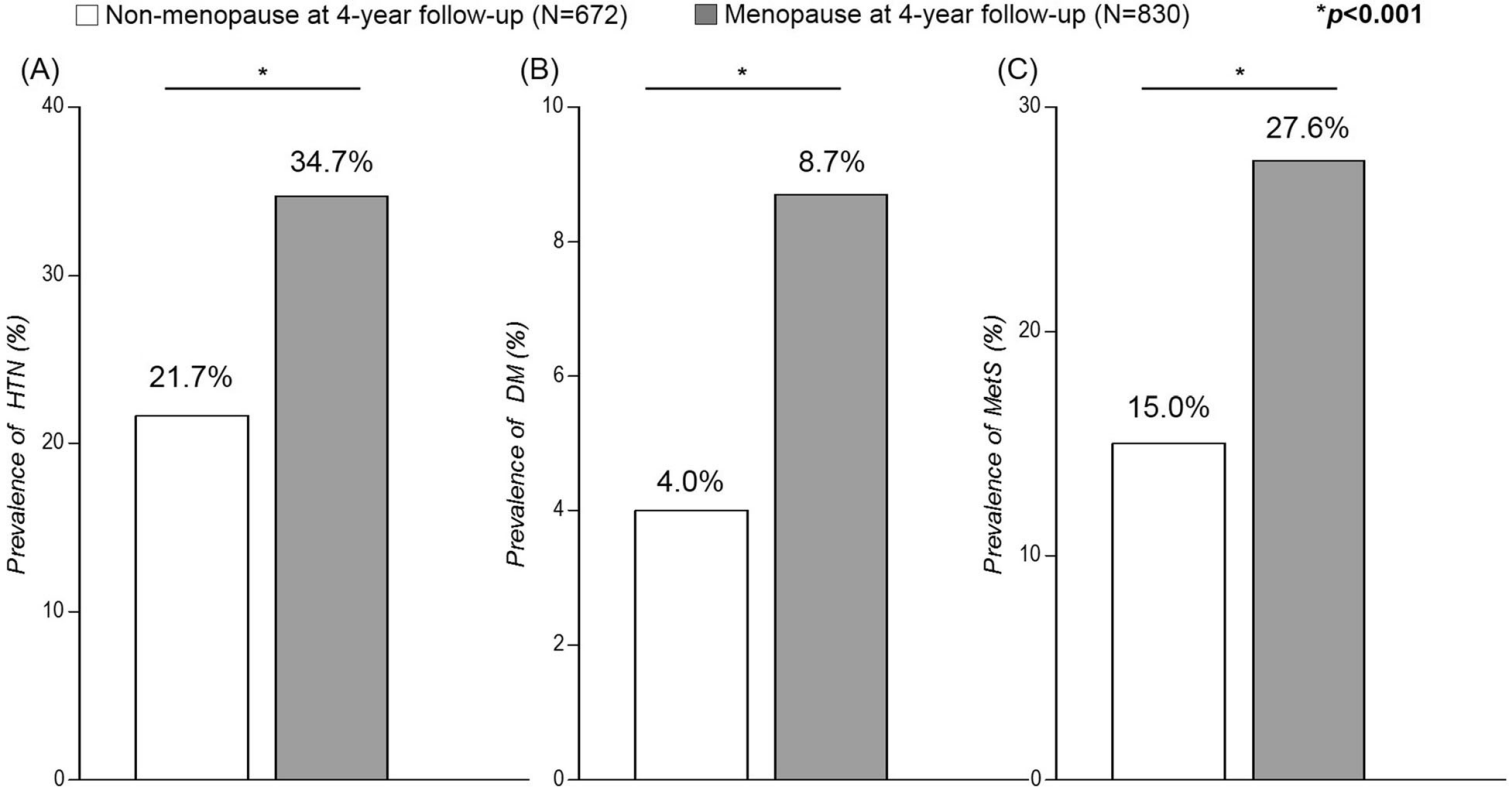
Change in prevalence of (**a**) hypertension, (**b**) diabetes mellitus, and (**c**) metabolic syndrome at 4-year follow-up in initially non-hypertensive patients. HTN, hypertension; DM, diabetes mellitus; MetS, metabolic syndrome

After 4 years, subjects who underwent menopause and those who were menopause at baseline showed a higher incidence of hypertension compared to non-menopausal subjects (16.3%, 13.1%, and 10.1%, respectively, *p* = 0.032) (Fig. 4a). The prevalence of diabetes and MetS were also higher in menopausal subjects at follow-up (Fig. 4b, c).

### Risk factors for development of hypertension

Univariate logistic regression analysis performed on the whole study population showed that age (OR 1.07, 95% CI 1.01–1.12), undergoing menopause during the study period (OR 1.73, 95% CI 1.14–2.62), increased BMI (OR 1.16, 95% CI 1.10–1.22), and MetS (OR 5.08, 95% CI 3.38–7.64) were associated with an increased risk of developing hypertension. However, multivariate regression analysis using the same variables showed that only BMI (OR 1.09, 95% CI 1.03–1.16) and MetS (OR 3.90, 95% CI 2.51–6.07) were significantly associated after adjustment (Table 3).

**Table 3:** Risk for development of hypertension

Univariate analysis	OR	95% CI	Multivariate analysis	OR	95% CI
Age	1.065	1.011–1.121	Age	1.040	0.971–1.115
Menopause after 4 years	1.731	1.143–2.622	Menopause after 4 years	1.391	0.863–2.242
Menopause at baseline	1.339	0.888–2.019	Menopause at baseline	0.965	0.565–1.647
BMI	1.157	1.097–1.220	BMI	1.093	1.031–1.159
MetS	5.080	3.377–7.642	MetS	3.736	2.396–5.827

*OR* odds ratio, *CI* confidence interval, *BMI* body mass index, *MetS* metabolic syndrome

## Discussion

Menopause is widely acknowledged to be associated with increased prevalence of hypertension in peri-menopausal women. However, our analysis suggests that increased prevalence may be attributable to increased BMI or presence of MetS, rather than menopause itself. Cross-sectional analysis at baseline and 4-year follow-up showed higher values of systolic and diastolic blood pressure in menopausal women. Moreover, most components constituting the modified NCEP-ATPIII criteria for MetS were more inclined to be positive in menopausal than non-menopausal subjects. In a longitudinal observation of subjects who were non-hypertensive at baseline, being menopause at the end of the period was associated with higher prevalence of hypertension, diabetes, and MetS. However, according to multivariate analysis assessing for risk of hypertension, the effect of menopause was insignificant after adjusting for other variables such as age. This finding suggests that the popular belief that menopause itself is a factor in development of hypertension in peri-menopausal women might not always be true. On the other hand, increased BMI and prevalence of MetS were associated with a significantly increased risk for development of hypertension, implying that not menopause per se, but increased BMI and MetS may be important factors behind hypertension. Controlling obesity and glucose tolerance, which are key factors of MetS, could be the key in preventing hypertension in this specific population.

There have been contradicting opinions on the role of menopause in development of hypertension. Historically, age, increased BMI, and menopause have been suggested as significant risk factors for hypertension in peri-menopausal women [23, 24]. Staessen et al. emphasized menopause as an important contributor of hypertension even after adjusting for age and BMI [5]. Their studies suggested that menopause was related to systolic, rather than diastolic hypertension, with the hypothesis that menopause decreased the compliance of large arteries through decrease in estrogen [6]. Findings of Zanchetti et al. also supported the effect of menopausal event on hypertension [7]. However, Casiglia et al. on the other hand, maintained that the effect of menopause on hypertension became negligible when adjusted for age [8]. Portaluppi et al. reported that increased BMI and older age, but not menopause were risk factors for hypertension in women near menopausal age [24]. Our study results suggest that increased BMI and prevalence of MetS are significant risk factors of developing hypertension in peri-menopausal women. Previous studies have also sought to address the relationship between obesity, menopause and hypertension, but their approach toward obesity was limited. Although BMI calculation is the simplest and most common way to evaluate obesity, it does not capture the metabolic and structural changes behind obesity such as dyslipidemia and abdominal obesity, which are frequently prevalent near menopausal age [25–27]. As there are evidence that central obesity is an independent risk factor of cardiovascular disease regardless of overall obesity [28], we used the prevalence of MetS to embrace the metabolic changes as a potential risk factor of development of hypertension in peri-menopausal women.

Pathogenesis of MetS has close relationships with visceral obesity and insulin resistance, which are key mechanisms that increase your blood pressure. Visceral obesity is suggested to cause hypertension via various mechanisms. Abdominal visceral fat increases sympathetic nerve activity in muscles [29]. Visceral fat also serves as an endocrine organ releasing adipocytokines such as leptin, resistin, TNF-α, and IL-6. These factors contribute to vascular inflammation and atherosclerotic changes [30], which also leads to increased blood pressure. Increased oxidative stress and decreased vasodilation are also attributable to an increase in visceral fat [31]. In addition, insulin has vasodilatory functions and aids the kidney in reabsorbing sodium. Insulin resistance has negative effects in vasodilation, but retains its renal effect, thereby further elevating blood pressure [32].

In our KoGES cohort, the prevalence of MetS was 15.0% for non-menopausal and 27.6% for menopausal subjects. According to the 2005 Korean National Health and Nutrition Examination Survey (KNHANES), the prevalence of MetS was 14.5% for women aged 40–49 and 37.8% for those aged 50–59 [33]. As the prevalence of MetS is similar in the KoGES cohort and the KNHANES 2005, the results of this study may be applicable to the general Korean population.

Additionally, data from KNHANES 1998–2008 show that the prevalence of low HDL-cholesterol in the overall population increased to over 60% in the Korean population. Furthermore, low HDL-cholesterol levels were more frequent in females than in males [34]. Our findings with the female population of KoGES are consistent with this general trend. The average level of HDL-cholesterol was lower than 50 mg/dL in both non-menopausal and menopausal subjects in our cohort. Kim et al. also reported that presence of low HDL-cholesterol was the most frequent component of MetS in Korean females, but was not significantly associated with menopause after adjusting for age [14].

Our study has several limitations. First, menopausal status was self-reported and data for more objective and detailed assessment of menopause were missing in the KoGES registry. Levels of serum FSH were not measured, and questionnaires did not include information about gynecologic operations such as hysterectomy or oophorectomy. Secondly, for many of the subjects, information on the use of anti-hypertensive medications were unavailable. Thus, we only focused on the prevalence of hypertension, rather than changes in SBP and DBP values. We also excluded subjects who did not provide required data and performed analysis on a per protocol set, which could have resulted in selection bias. Lastly, hormonal changes during the peri-menopausal period can bring about metabolic syndrome, but also has effects on the renin-angiotensin system and affects production of angiotensin and sodium metabolism, which in turn can also lead to hypertension [31, 35]. Our study suggests that metabolic syndrome has a positive relationship with hypertension in peri-menopausal women, but the complex mechanism to hypertension could not be fully assessed in our analysis.

The finding that MetS is a risk factor for development of hypertension in peri-menopausal women has clinical implications. Menopause is an aging process and is closely related with central obesity and development of MetS. However, we may be able to prevent the development of other co-morbidities such as hypertension, if we are able to control MetS. Reducing excessive energy intake and enhancing physical activity is needed to deal with hypertension near menopausal age. Our study provides the evidence for more strict monitoring and treatment of central obesity and insulin resistance in this specific population.

## Conclusions

Closely monitoring and treating metabolic syndrome in women near menopause could help prevent hypertension.

## Additional file

Additional file 1: Table S1. Characteristics of study subjects according to menopausal status at 4-year follow-up. (DOCX 19.4 KB)
