# Supplementary material for: Metabolic syndrome, not menopause, is a risk factor for hypertension in peri-menopausal women
Source: Clin Hypertens. 2018 Oct 15;24:14. doi: 10.1186/s40885-018-0099-z (PMC6191993; doi:10.1186/s40885-018-0099-z)
Supplement: Supplementary file 1 — Table S1. Characteristics of study subjects according to menopausal status at 4-year follow-up. (DOCX 19.4 KB) [file 40885_2018_99_MOESM1_ESM.docx]

Additional file 1: Table S1. Characteristics of study subjects according to menopausal status at 4-year follow-up

|  | Non-menopause  (N=672) | Menopause  (N=830) | *P*-value |
| --- | --- | --- | --- |
| Age (years) | 48.2 ± 2.0 | 52.3 ± 3.1 | <0.001 |
| Weight (kg) | 59.2 ± 7.9 | 59.5 ± 7.8 | 0.450 |
| Height (cm) | 155.6 ± 5.2 | 155.0 ± 5.2 | 0.033 |
| BMI (kg/m^2^) | 24.4 ± 3.1 | 24.7 ± 3.0 | 0.062 |
| SBP (mmHg) | 109.1 ± 14.2 | 113.6 ± 16.3 | <0.001 |
| DBP (mmHg) | 74.0 ± 10.4 | 76.9 ± 10.0 | <0.001 |
| Waist circumference (cm) | 79.7 ± 8.4 | 82.5 ± 8.8 | <0.001 |
| TG (mg/dL) | 106.0 ± 70.5 | 131.1 ± 77.1 | <0.001 |
| Total cholesterol (mg/dL) | 188.8 ± 31.6 | 202.8 ± 35.7 | <0.001 |
| HDL-cholesterol (mg/dL) | 47.3 ± 10.4 | 46.1 ± 10.2 | 0.033 |
| Fasting glucose (mg/dL) | 88.4 ± 11.0 | 90.2 ± 14.6 | 0.007 |
| HbA1c (%) | 5.4 ± 0.4 | 5.5 ± 0.5 | <0.001 |
| Alcohol consumption (%) | 35.9 | 27.2 | <0.001 |
| Hypertension prevalence (%) | 21.7 | 34.7 | <0.001 |
| Diabetes prevalence (%) | 4.0 | 8.7 | <0.001 |
| MetS prevalence (%) | 15.0 | 27.6 | <0.001 |

BMI, body mass index; SBP, systolic blood pressure; DBP, diastolic blood pressure; TG, triglyceride; MetS, metabolic syndrome. Data presented as mean ± SD for continuous variables and percentages for categorical variables
